# Adverse psychiatric effects of lorlatinib: a case report and review of the literature

**DOI:** 10.1192/j.eurpsy.2025.1788

**Published:** 2025-08-26

**Authors:** I. Ortín, L. Nocete

**Affiliations:** 1 Virgen Macarena University Hospital, Seville; 2 La Paz University Hospital, Madrid, Spain

## Abstract

**Introduction:**

Lung cancer is one of the most frequent forms of cancer worldwide. Its prevalence has increased substantially the last decades. About 85% of all cases are non-small-cell lung cancer, which can be further divided in other subtypes. The use of targeted therapies and immune check-point inhibitors in the last years has improved survival, such is the case of tyrosin-kinasa inhibitors as lorlatinib. This molecule can reduce neoplastic growth when ALK rearrangement is present.

**Objectives:**

To review the literature about psychiatric adverse effects of lorlatinib.

**Methods:**

A narrative review of the evidence was performed. The search was focused on described psychiatric adverse effects in patients with lung cancer who have received lorlatinib as antineoplastic agent.

**Results:**

We report the case of a 54-year-old man with lung adenocarcinoma stage IV ALK+ diagnosed in 2024. Suprarenal metastases were found. He started antineoplastic treatment with lorlatinib as first line agent during hospitalization, with a dose of 100mg per day. Three months later, the patient experienced anxiety and impulse phobias of aggressive content which led to an intense psychological distress and behavioural repercussion (avoidance, overcompensation). He consulted because of these symptoms and the dose of lorlatinib was reduced to 75mg per day by his oncologist. By the time he was evaluated in Psychiatry Service, the symptoms had improved but remained present. Low doses of aripiprazole were prescribed as symptomatic treatment (2,5mg per day) with great results manifested one week later.

**Conclusions:**

Most common adverse effects of tyrosin-kinasa inhibitors are fatigue, gastrointestinal symptoms, skin reactions or edema, among others. Although neuropsychiatric adverse effects are infrequent, they can occur and induce significative impairment and emotional distress. It’s important to recognize these other symptoms, especially on new drugs, and know how to approach them properly.

**Disclosure of Interest:**

None Declared

